# Genetic effects on coat colour in cattle: dilution of eumelanin and phaeomelanin pigments in an F2-Backcross Charolais × Holstein population

**DOI:** 10.1186/1471-2156-8-56

**Published:** 2007-08-16

**Authors:** Beatriz Gutiérrez-Gil, Pamela Wiener, John L Williams

**Affiliations:** 1 Roslin Institute (Edinburgh), Roslin, Midlothian, Scotland, UK Midlothian EH25 9PS, UK; 2 Current Address: Parco Tecnologico Padano, Via Einstein, Polo Universitario, Lodi 26900, Italy

## Abstract

**Background:**

In cattle, the gene coding for the melanocortin receptor 1 (*MC1R*) is known to be the main regulator of the switch between the two coat colour pigments: eumelanin (black pigment) and phaeomelanin (red pigment). Some breeds, such as Charolais and Simmental, exhibit a lightening of the original pigment over the whole body. The dilution mutation in Charolais (*Dc*) is responsible for the white coat colour of this breed. Using an F2-Backcross Charolais × Holstein population which includes animals with both pigment backgrounds, we present a linkage mapping study of the Charolais dilution locus.

**Results:**

A Charolais × Holstein crossbred population was investigated for genetic effects on coat colour dilution. Three different traits representing the dilution of the phaeomelanin, eumelanin, and non-pigment-specific dilution were defined. Highly significant genome-wide associations were detected on chromosome 5 for the three traits analysed in the marker interval [ETH10-DIK5248]. The *SILV *gene was examined as the strongest positional and functional candidate gene. A previously reported non-synonymous mutation in exon 1 of this gene, *SILV *c.64A>G, was associated with the coat colour dilution phenotype in this resource population. Although some discrepancies were identified between this mutation and the dilution phenotype, no convincing recombination events were found between the *SILV *c.64A>G mutation and the *Dc *locus. Further analysis identified a region on chromosome 28 influencing the variation in pigment intensity for a given coat colour category.

**Conclusion:**

The present study has identified a region on bovine chromosome 5 that harbours the major locus responsible for the dilution of the eumelanin and phaeomelanin seen in Charolais crossbred cattle. In this study, no convincing evidence was found to exclude *SILV *c.64A>G as the causative mutation for the Charolais dilution phenotype, although other genetic effects may influence the coat colour variation in the population studied. A region on chromosome 28 influences the intensity of pigment within coat colour categories, and therefore may include a modifier of the *Dc *locus. A candidate gene for this effect, *LYST*, was identified.

## Background

As in many mammals, coat colour in cattle results from the relative presence of eumelanin (black-brown pigment) and phaeomelanin (red-yellow pigment), the two basic pigments produced by melanocyte cells [1]. Pigment production takes place in the melanosomes, organelles containing the enzymes directly involved in pigment biosynthesis. Tyrosinase (TYR) is the rate-limiting enzyme in the melanogenesis pathway. High levels of this enzyme are required for the production of eumelanin, whereas low enzyme levels result in the production of phaeomelanin [2,3]. Tyrosinase activity is regulated by the melanocortin 1 receptor (MC1R or α-MSHR), whose stimulation by α-melanocyte-stimulating hormone (α-MSH) leads to the production of eumelanin [4]. Phaeomelanin is produced in absence of α-MSH stimulation, either as result of a non-functional MC1R receptor [5] or in the presence of the Agouti protein, which is secreted by cells adjacent to melanocytes and acts as an antagonist of the α-MSH action by blocking the MC1R [6]. In addition to the genes coding for these proteins essential for pigmentation, work in mice has uncovered more than 120 genes involved in colour variation. These include genes involved in the biosynthesis of melanin (*Tyrp1*, *Tyrp2*), the biology of melanocytes and melanosomes (e.g. *ePomc1*, *Mitf*, *Silver*, *Ap3*, *Mlph*, *Myo5a*, *Rab27a*) and migration and survival of melanocytes during development (e.g. *Kit*, *Kitl*, *Edn3 and Ednrb*) [7]. This information provides a number of candidate genes that may also affect coat colour in other species, including cattle.

In cattle, the *Extension *locus (*MC1R*), located on chromosome 18 [8], plays a major role in the regulation of the synthesis of eumelanin versus phaeomelanin. The most common alleles at this locus are the dominant *E*^*D *^allele and the recessive *e *allele, which are responsible for the black and red colour, respectively, and code for a receptor which is not affected by the Agouti protein. Breeds with a mix of red and black hairs (wild-type colour) carry Agouti-receptive alleles (*E*^+^, *E*^1 ^and *E*^2^) [9,10]. In contrast to mice, the role of the bovine locus for the Agouti protein (ASIP) in colour variation seems limited as no allelic variants have been found in the coding sequence [11].

In some cattle breeds, such as Dexter, Galloway, Charolais, Highland and Simmental, a lightening or dilution of the base colour defined by the *Extension *locus is observed. A wide range of colours results from this dilution phenomenon (white, cream, dun, gold, yellow, pale red, grey or brown). The gene responsible of the dilution seems to vary between breeds: e.g. the pale (dun) coat colour observed in Dexter cattle is due to the gene coding for the TYRP1 (tyrosine related protein 1), but this gene has been excluded as being responsible for dilution in other breeds [12]. The silver (*SILV*) gene, which codes for a type I integral membrane protein in the pre-melanosome matrix (PMEL17) [13], and which is essential for melanosome development [14,15], has been found to be responsible for coat colour dilution in Highland cattle [16].

The Charolais breed exhibits the most extreme case of dilution, as pure-bred Charolais individuals have a uniform white coat colour, despite the *e/e Extension *genotype of most Charolais cattle. Therefore, the characteristic coat colour of this breed results from a phaeomelanin dilution over the entire body. Charolais cattle are thought to be homozygous for a dilution mutation (Dilution Charolais, *Dc/Dc*), that in heterozygotes (*Dc/dc*^+^) produces an intermediate phenotype: grey colour if the dilution applies to eumelanin (e.g. in a Black Angus × Charolais cross) or pale red or yellow if the background pigment is phaeomelanin [17].

Using an F2 Holstein × Charolais population, Kühn and Weikard [18] recently reported an association between dilution of black pigment (eumelanin) and a region on bovine chromosome 5 including the *SILV *gene. However, these authors did not include animals of red background in their analysis, and therefore it remains to be shown whether the locus affecting the dilution of phaeomelanin, typical of the Charolais breed, co-locates with the linkage association reported by these authors.

We performed a genome scan to localise the genetic locus responsible for the dilution phenotype using a Charolais × Holstein experimental population obtained through a F2 and balanced Backcross design. The linkage analysis included individuals with both black and red coat colour background, and therefore addressed the localization of the major locus involved in the dilution of the phaeomelanic pigment in the Charolais breed.

## Results

### Pedigree and Phenotypic Data distributions

A total of 436 animals were scored for coat colour. These included 273 F2 individuals and 163 reciprocal backcrosses (77 Charolais backcrosses, CB1, and 86 Holstein backcrosses, HB1).

**Figure 1:**
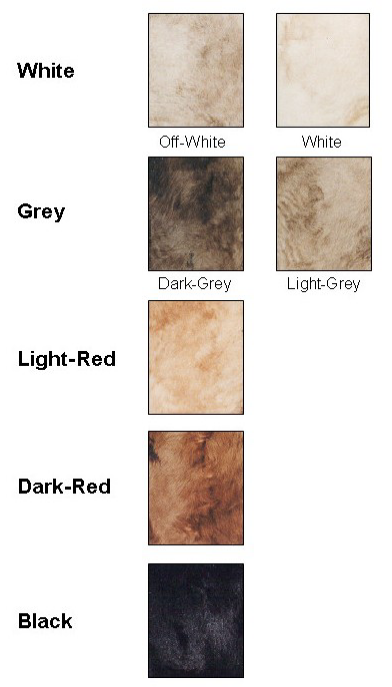
**Coat colour categories used for visual scoring of the second-generation individuals of the F2-Backcross population studied**. The primary analysis was based on the five category colour scoring (White, Grey, Light-Red, Dark-Red and Black). The initial visual scoring had considered seven subcategories (White, Off-White, Light-Grey, Dark-Grey, Light-Red, Dark-Red and Black).

The number of individuals scored in each of five colour categories (White, Grey, Light-Red, Dark-Red and Black; See Figure 1) is detailed in Table 1A. The phenotypic proportions observed for the CB1, HB1 and F2 were consistent with the assumption of fixation of alternative alleles at the Dilution locus in the Charolais and Holstein founders. A total of 141 individuals were included in the analysis of the Grey-Intensity trait, with 91 individuals scored as Light-Grey and 50 as Dark-Grey. The number of animals included in these two sub-categories for each genetic background group is detailed in Table 1B.

**Table 1:** Distribution of phenotypic coat colour scores across the genetic background groups. Number of animals of each genetic background group (F2, HB1 and CB1) scored in each of the defined coat colour categories (A) and subcategories (B) included in the combined traits analyzed in this study.

**Colour Scoring Categories (inferred *Dc *locus genotype)**	**Description**	**CB1**	**F2**	**HB1**	**Total**
**A) Coat colour categories included in the dilution-related traits**					
Black (*dc*^+^/*dc*^+^)	Dark brown, black	0	43	37	**80**
Dark-Red (*dc*^+^/*dc*^+^)	Reddish, dark-red	0	23	1	**24**
Light-Red (*Dc*/*dc*^+^)	Yellowish or pale red	23	36	1	**60**
Grey (*Dc*/*dc*^+^)	Greying or Brownish	15	93	47	**155**
White (*Dc*/*Dc*)	White colour	39	78	0	**117**
Total		**77**	**273**	**86**	**436**
**B) Subcategories included in the Grey-Intensity trait**					
Dark-Grey (*Dc*/*dc*^+^)		3	24	23	**50**
Light-Grey (*Dc*/*dc*^+^)		8	63	20	**91**
Total		**11**	**87**	**43**	**141**

A REML analysis showed the genetic background (F2, CB1 and HB1) to have a significant influence (p < 0.001) on the five category colour scores. Other variables, such as birth year and sex, did not show significant influences on the coat colour.

### Genotypes at the *MC1R *locus

The genotyping of the *MC1R *showed that the founder lines were almost fixed for alternative alleles at the *Extension *locus, with all the Charolais sires being *ee*, and 90% of the Holstein dams being *EE *genotype. The remaining 10% of the Holsteins dams were either *E*^*D*^*E*^+ ^or *Ee *(approximately half of each), and one had the recessive genotype *ee*. The second-generation individuals classified as White included the most common *MC1R *genotypes (*E*^*D*^*E*^*D*^, *Ee*, *ee*). For the other coat colour categories, the *Extension *locus genotypes were in agreement with the colour score assigned to the animals (red = *ee *and black = *E*^*D*^-). The *E*^+ ^allele was rare, with only three individuals with *E*^*D*^*E*^+ ^genotype (two greys and one black) and another three with *E*^+^*e *genotype (two reds, light and dark, and one grey).

### Linkage analysis results for the eumelanin and phaeomelanin dilutions

#### Evidence for a major gene effect

The initial analysis for the binary colour traits (White, Grey, Light-Red, Dark-Red and Black) showed highly significant effects on chromosomes 5 and chromosome 18 (data not shown). The colour categories were subsequently grouped to give combined traits related to the dilution phenotype: Quantitative-Dilution, Quantitative-Black and Quantitative-Red.

**Figure 2:**
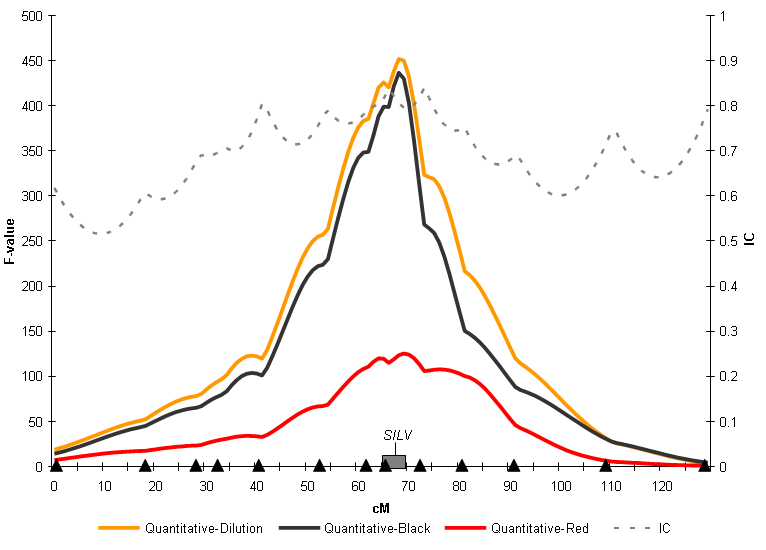
**Linkage evidence on bovine chromosome 5 for the locus affecting the eumelanin and phaeomelanin dilution in Charolais crosses**. F-ratio profile obtained for the dilution-related traits Quantitative-dilution, Quantitative-Black and Quantitative-Red on chromosome 5. Dashed lines indicate information content (right *y*-axis). Marker positions are identified as triangles above the *x*-axis. The peak of the statistical profiles was flanked in all the cases by markers ETH10 and DIK5248. The bootstrap 95% CI for Quantitative-Black is indicated as a grey box above the *x-*axis. The position of the *SILV *gene according to our linkage analysis is represented on the x-axis.

The results of the regression analysis revealed genome-wide significant associations for the three dilution-related traits on chromosome 5 (Table 2), with the peak of the statistical profiles observed at positions 68 (for Quantitative-Black and Quantitative-Dilution) and 69 cM (for Quantitative-Red) on the linkage map, between markers EHT10 and DIK5248 (Figure 2). The number of individuals included in the analyses, differed between the three traits (Table 2), which may explain the differences in the significance levels and size of effects obtained. The size of the 95% confidence interval calculated with respect to these linkage associations ranged between 5–6 cM (for Quantitative-Black and Quantitative-Dilution) and 19 cM (for Quantitative-Red). The average QTL position calculated by bootstrapping for the three traits was between 68.11 and 68.40 cM.

**Table 2:** Significant associations detected for the dilution-related traits and the Grey-Intensity trait. For each significant association, the position, and gene effect estimates (additive and dominance) are detailed. See Methods for further details about the trait categories.

**Chrom**.	**Trait**	**Position (cM)**	**F-value**	**p_c_-value**	**Additive Effect**	**Dominance Effect**
**5**	Quantitative-Dilution^▲^	68	452.0	<0.0001	0.93 ***	-0.06
**5**	Quantitative-Black	68	436.6	<0.0001	0.94 ***	-0.11 *
**5**	Quantitative-Red	69	125.1	<0.0001	0.86 ***	-0.14
**28**	Quantitative-Dilution^▲^	0	4.9	0.016	-0.19 **	0.00
**28**	Quantitative-Red	0	5.9	0.019	-0.39 ***	0.18
**28**	Grey-Intensity^▲▲^	1	5.3	0.016	-0.12	-0.32 **

Quantitative-Dilution (443 individuals included in analysis): White (1), Pale colour (2; Light-Red and Grey), Dark colour (3; Dark-Red and Black).

Quantitative-Black (327): White, only whites of *E*^*D*^- genotype at *MC1R *(1), Grey (2), Black (3).

Quantitative-Red (130): White, excluding whites of *E*^*D*^- genotype at *MC1R *(1), Light-Red (2), Dark-Red (3).

Grey-Intensity (141): Light-Grey (1), Dark-Grey (2)

p_c_-value: chromosome-wide p-value

* p-value < 0.05; ** p-value < 0.01; ** p-value < 0.001

^▲ ^Includes *MC1R *genotype (*E*^*D*^-, *E*^+^*e*, *ee*) as a fixed effect.

^▲▲ ^Includes *SILV *c.64A>G genotype as a fixed effect.

For the three dilution-related traits, the signs and magnitudes of the additive and dominance estimates indicated that two copies of the Holstein allele would make the animal dark (Black or Dark-Red), whereas two copies of the Charolais allele would produce a white animal. For the three traits, the additive effects were much higher than the negative dominance effects, indicating that, in most of the cases, the heterozygotes were scored as intermediate phenotype (Grey or Light-Red). The slight dominance of the Charolais allele, which was significant only for Quantitative-Black, indicates that some heterozygotes were scored as White.

The *SILV *gene, which has a known function in the production of pigmentation in the melanocytes [14,15], is included in the confidence interval of these significant associations (Figure 2), and therefore appears as a strong candidate. A non-synonymous mutation located in the first exon of this gene (*SILV *c.64A>G) at position 64 of the coding region [GenBank: EF065525] has been found exclusively in the Charolais breed, and suggested as possibly being the causative mutation for the coat colour dilution characteristic of this breed [19]. We genotyped this mutation across the second-generation individuals and tested the possible association of this mutation with the significant effects identified on chromosome 5. When the *SILV *c.64A>G mutation was included in the regression model as a fixed effect, the genome-wide significant effects initially identified for the dilution-related traits on chromosome 5 were no longer significant.

#### Minor gene effects

In addition to the large effect on chromosome 5, the initial analysis identified linkage associations on chromosome 28 for both Quantitative-Dilution and Quantitative-Red that exceeded the suggestive linkage threshold defined by Lander and Kruglyak [20], which for the bovine genome corresponds to a chromosome-wide p-value < 0.034 (Table 2). These associations showed a significant additive effect, with the negative sign indicating that the Charolais allele at this locus increased the presence of pigment. When the *SILV *c.64A>G variant was added to the model as a fixed effect, chromosome-wide significant effects were also detected on chromosomes 1 and 15 for Quantitative-dilution and Quantitative-Black, respectively, although neither had significant additive effects (results not shown).

The analysis of the Grey-Intensity revealed one significant association at the proximal end of chromosome 28 (Table 2), mapping to the same location as the associations identified by the initial analysis for Quantitative-Dilution and Quantitative-Red. However, only the dominance effect was significant for this association.

### Genotypes for results of the *SILV *c.64A>G mutation

Genotypes for the *SILV *c.64G>A mutation were obtained for the majority of animals and the distribution of genotypes for this allelic variant in relation to the colour score is shown in Table 3A. A REML analysis identified a significant association (p-value < 0.001) between phenotype and genotype for this mutation. The segregation at the *SILV *c.64A>G locus in the three genetic background groups (Table 3B) did not deviate significantly from the expected proportions assuming fixation of this mutation in the founder lines ("A" in Charolais and "G" in Holsteins). The genotypes of the *SILV *c.64A>G mutation were later included in the linkage analysis of chromosome 5 (assuming fixation of alleles in the founder lines), with this dinucleotide marker mapping at position 67.3 cM of the chromosome 5 linkage map, between markers ETH10 and DIK5248 [see Additional file 1].

**Table 3:** Genotypes of the *SILV *c.64A>G mutation in the F2-Backcross population considered in this study. Distribution of genotypes across the five colour categories defined in this work (A) and across the three genetic background groups of the studied population, CB1, F2 and HB1 (B). Numbers in bold indicate the discrepancies observed in our dataset between the *SILV *c.64A>G genotypes and the dilution phenotype.

	**AA**	**AG**	**GG**	**Total**
**A) Colour score categories**				
**Black**			80	80
**Dark-Red**		**1**	21	22
**Light-Red**	**9**	52		61
**Grey**	**6**	145	**1**	152
**White**	103	**13**		116
**B) Genetic backgroundgroups**				
**CB1**	41	34		75
**F2**	77	132	63	272
**HB1**		45	39	84

**Figure 3:**
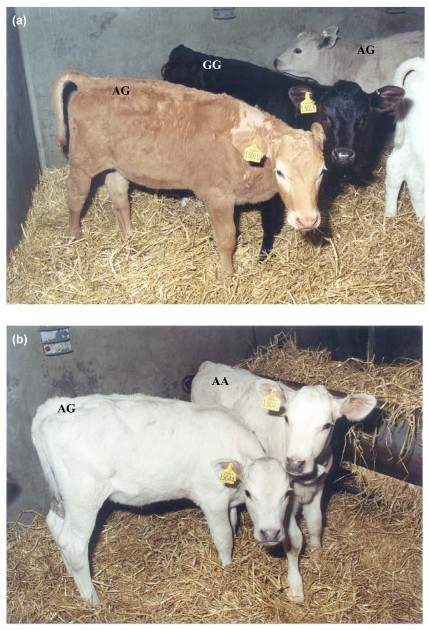
**Examples of dilution phenotypes observed in the F2-Backcross individuals**. **A. Calves with partially diluted red and black coat colour background (scored as Grey and Light-Red) together with a non-diluted individual (scored as Black)**. The *SILV *c.64A>G mutation showed significant association with the dilution phenotypes (p-value < 0.001). **B. Two calves showing completely diluted phenotypes (scored as White)**. For the animal on the left, the genotype of the *SILV *c.64A>G mutation was not consistent with the presumed genotype at the *Dc *locus. For discrepancies like this, however, no convincing recombination between *SILV *c.64A>G and the *Dc *locus was observed.

Animals carrying one or two copies of the A allele (GA and AA) showed, in general, a partial or complete dilution, respectively (Figure 3A). However, there were a few individuals, about 7% of the total, for which the genotype of this mutation could not be used to predict the colour category in which the individuals had been included by visual scoring (indicated in bold in Table 3A), as shown in Figure 3B.

To further investigate the nature of discrepancies between the actual phenotype and that predicted by *SILV *c.64A>G genotype, we subsequently included the putative *Dc *locus in the linkage analysis. With this aim, the genotypes of the *Dc *locus for the second generation animals (F2, CB1 and HB1) were ascertained based on the phenotypic colour score (dark colour: *dc*^+^*dc*^+^; pale colour: *Dcdc*^+^; white colour: *DcDc*) based on the previously-documented mode of inheritance for this locus [17]. This analysis positioned the *Dc *locus in the same marker interval as *SILV *c.64A>G (recombination fraction, θ = 0.04). For the animals showing discrepancies, as described above, a CHROMPIC analysis did not suggest any genotype errors for the *SILV *c.64A>G mutation. However, the *Dc *locus was involved in unlikely double recombination events (resulting in θ > 0) for most of these animals, suggesting either phenotype scoring errors or the influence of other genetic effects influencing the phenotype of these animals. Four discordant animals showed a putative single recombination event between the *SILV *c.64A>G and the *Dc *locus.

### Sequencing analysis results

The coding region of the bovine *SILV *gene was sequenced to detect polymorphisms, using as a reference the published mRNA sequence [GenBank: EF065525]. In addition to the *SILV *c.64A>G mutation in exon 1, a previously unreported substitution in exon 2 was identified that affects the second residue of codon 36 (c.107G>T), causing an amino acid change from serine to leucine. A T>C substitution in intron 2 (c.187+56T>C) was also identified. The other polymorphisms observed have been previously reported: (i) a silent mutation in exon 6 affecting the third residue of the codon 374 (c.1122C>A) [18]; (ii) a non-synonymous mutation in exon 11 affecting the second nucleotide of codon 612 (c.1835C>A) [GenBank EF363685], leading to an alanine for glutamic acid substitution. Apart from the c.64A>G mutation, none of these allelic variants were associated with the dilution phenotype of the 16 individuals analyzed.

## Discussion

The variation observed in the coat colour of the F2 and Backcross individuals of this Charolais × Holstein population provides an opportunity to investigate effects and the mode of inheritance of the Charolais dilution locus. Based on the phenotypic data (Table 1), it is clear that the *Dc *and the *Extension *loci are mainly responsible for the variation in coat colour observed. As multiple alleles at the *Extension *(*MC1R*) locus were segregating in this population, the effect of the *Dc *locus on both types of backgrounds was confirmed by the observation of a complete or partial dilution affecting individuals with *E*^*D*^-, *E*^+^*e *or *ee MC1R *genotype. The pale colour observed in individuals with *E*^+^*e MC1R *genotype demonstrates the dilution of pigments produced by both Agouti-responsive (*E*^+^-) and non-responsive (*ee*) melanocytes. This consistency of the effect across *MC1R *genotypes was also supported by the results of the genome scan, in which the same region on chromosome 5 showed linkage with the three dilution-related traits analyzed. In addition, the additive effects estimated for Quantitative-Black and Quantitative-Red had similar size. The dominance effect was very small relative to the additive effect; therefore a single copy of the *Dc *allele originating from the Charolais is sufficient to dilute either eumelanin or phaeomelanin. Heterozygous individuals, *Dc/dc*^+^, are generally of intermediate phenotype (light-grey or light-red) and two copies of the *Dc *allele are required to produce a complete dilution of the original pigment (white phenotype). These results are consistent with the inheritance of the Charolais dilution locus described in the literature [17]. The data also support the assumption of alternative fixed alleles at the *Dc *locus in the founder lines, on which the regression analysis was based. Therefore, the power of detection of this locus was maximised, which is reflected in the high significance of the associations identified on chromosome 5.

The location of the major gene associated with the Quantitative-Dilution, Quantitative-Black and Quantitative-Red traits is coincident with the position reported in a linkage study of eumelanin dilution (black pigment) in a Holstein × Charolais F2 population [18]. In the region of bovine chromosome 5 flanked by markers ETH10 and DIK5248, there are several metabolic candidate genes directly related to pigmentation pathways (*ErbB3*, *SILV*), and members of gene families where at least one member is suggested to have an effect on pigmentation (*BLOC1S1*, *RAB5b*, *DCTN2 and MYO1A*). Among these, the *SILV *gene is the only one with an established function in the melanocyte and therefore is the strongest candidate. This gene is between 56.407 and 56.415 Mb in the latest version of the bovine genome sequence assembly (Build 3.1) [21], according to which, ETH10 is located at 55.333 Mb [21]. It codes for a pre-melanosomal matrix protein (PMEL17) necessary for the formation of the fibril matrix upon which melanin intermediates are deposited late in melanosome maturation [14]. Mutations in the *SILV *gene are known to cause diluted phenotypes in mice [22], horse [23] and dog [24], although in these species the effect is to block the production of eumelanin without effects on phaeomelanin. In chicken, allelic variations in this gene also block the production of black pigment in the plumage leading to the *Smoky, Dun*, and *Dominant white *colour variants [25].

In cattle, a Charolais-specific allele has previously been reported in exon 1 of the bovine *SILV *gene [19]. This mutation is a G>A substitution that results in a change from glycine to arginine within the N-terminal signal sequence of the PMEL17 protein. Among thirteen breeds tested, the A allele was only identified in pure-breed Charolais individuals or Charolais crosses [19]. This mutation was genotyped across the individuals of the resource population scored for coat colour (F2, CB1 and HB1). The observed distribution of genotypes within the three genetic background groups supports the hypothesis of fixation of alleles in the founder lines, with the A allele only present in Charolais founders. To test the association between the *SILV *c.64A>G genotypes and the diluted phenotype, the genotype of this mutation was included as a fixed effect in the regression model fitted to the Quantitative-Dilution, Quantitative-Black and Quantitative-Red traits. For all traits, inclusion of this variant resulted in the disappearance of the highly significant linkage associations, suggesting that the *Dc *locus is either due to or in strong linkage disequilibrium with the *SILV *c.64A>G mutation.

Some discrepancies between *SILV *c.64A>G and the phenotype were observed however, which draws into question whether *SILV *c.64A>G is the causative mutation underlying the *Dc *dilution effect, as other authors have suggested [18]. For these discordant animals, the CHROMPIC analysis including the *SILV *c.64A>G mutation and the *Dc *locus (presumed genotypes based on phenotypes) did not suggest genotyping errors for the tested mutation, however, most of these animals appeared as double recombinants at the *Dc *locus. The probability of a genuine double recombination event in such a small chromosomal interval is very low, and to detect several such double recombinants in the number of animals examined here would be very unlikely. Hence, apart from possible phenotype-genotype mismatches, these double recombination events are more likely to be the result of either phenotypic mis-scoring or the effect of other loci influencing coat colour. Possible mis-scoring may be explained by difficulties in distinguishing between the partially and the completely diluted phenotypes (especially Light-Grey/Light-Red against Off-White) or in scoring some individuals showing a non-homogenous dilution along the body (e.g. darker head than body).

Under the possibility of another locus or loci affecting the coat colour variation in this population, the results of the analysis of Grey-Intensity may help to interpret the minor gene effects revealed by the primary analysis. The proximal region of chromosome 28 was the only significant effect at the suggestive level for the diluted-related traits and for the Grey-Intensity trait, which suggests that these significant associations could result from the true quantitative nature of coat colour intensity within and between the phenotype classes. This locus could, therefore, be considered as a candidate for the genetic background effects that underlie subtle variations in colour, and that in certain cases could lead to discordance between colour score and the *SILV *locus genotype (e.g. this could explain discrepancies such as AG animals that were scored as White). A colour-associated gene, LYST (lysosomal trafficking regulator), maps to the proximal end of chromosome 28 [26]. Mutations in this gene are responsible for Chediak-Higashi syndrome 1 in human and mouse (*beige *mutant). This disorder has been reported in Japanese black cattle [27] and is characterized by prolonged bleeding time and, more relevantly for this paper, a light coat colour. Our results indicate that allelic variation at this gene, possibly not associated with illness, could underlie the different shades of colours observed in the partially diluted colour categories by acting as a modifier of the *Dc *locus. Increased marker density in this chromosomal region would be required before an epistatic analysis between this locus and the *Dc *locus could be conducted.

Other genetic effects may be the result of the interaction of the causal mutation of the Charolais dilution phenotype and other mutations in the *SILV *gene. For instance, the AG individual with Dark-Red phenotype rather than the expected Light-Red may be explained if another mutation rescued the dilution effect due to the *SILV *c.64A>G mutation as seems to be the case with the *Smoky *phenotype in chickens, which in addition to the 9-bp deletion in exon 10 of the *SILV *gene associated with the *Dominant white *phenotype, also have an additional deletion in exon 6 that partially restores pigment production [25].

Based on the CHROMPIC analysis, only four discordant animals showed a putative single recombination event between the *SILV *c.64A>G and the *Dc *locus, however, these discrepancies could not be conclusively confirmed as the phenotype of these individuals was intermediate between pale (Light-Grey/Light-Red) and Off-White. Based on the lack of convincing recombinants between the *SILV *c.64A>G mutation and the *Dc *locus, this allelic variant of the *SILV *gene cannot be ruled out as the causal mutation of the Charolais dilution phenotype. The effect of this locus on the phenotype is supported by the loss of significance in the regression analysis when this mutation is included as a fixed effect in the model. However, this does not exclude the possibility of a different mutation tightly linked to *SILV *c.64A>G being the *Dc *causal mutation, although we and others [18] have not found other mutations associated with coat colour in the coding region of the *SILV *gene.

The interaction between the *SILV *gene and pigment type appears to be complicated. The pigment-specificity of mutations in the *SILV *gene observed in other species [22-25] is in agreement with the critical role reported for this protein in eumelanosomes but not in phaeomelanosomes [28] and the suppression of PMEL17 expression seen in murine phaeomelanosomes [29,30]. However, recent work in Highland cattle reported a 3-bp deletion in exon 1 of the bovine *SILV *gene associated with the dilution of both red and black pigments [16]. This finding, and the likely association of the *SILV *gene and the *Dc *locus, which affects both pigments, are intriguing and may suggest that the role of PMEL17 differs between species. This is plausible as the genuine function of the *SILV *gene product in pigmentation is not completely understood [14] and the biological basis of pigmentation may vary with species. Mutations in the *SILV *gene that have only been shown to affect eumelanin background are located in the c-terminal sequence of the *SILV *gene and affect the transmembrane or cytoplasmatic domains of the protein [22-25]. It is possible that mutations closer to the N-terminal end (such as exon 1, where both cattle mutations are found) could lead to more general interference with pigment production. Exon 1 codes for the signal peptide sequence of the protein [14], which is thought to determine the entry of PMEL17 into the secretory pathway prior to its processing and cleavage [31].

## Conclusion

The work reported in this paper localizes the locus responsible for the dilution effect of the Charolais breed, *Dc*, to bovine chromosome 5, and demonstrates that this locus acts on both black (eumelanin) and red (phaeomelanin) pigment backgrounds. The bovine *SILV *gene was assessed as a candidate for this linkage association. Although the non-synonymous *SILV *c.64A>G mutation, previously described [19], does not explain all the phenotypes in the population studied, no convincing evidence was found to exclude it as the causative mutation for the Charolais dilution phenotype. Other genetic effects, such as those observed on chromosome 28 for pigment intensity, may be influencing the coat colour variation of this population. A candidate gene for this effect, *LYST*, has been identified.

## Methods

### Animals and Phenotypes

A total of 137 F1 animals resulting from a cross between Charolais bulls with pure bred Holstein cows were used to generate 501 second-generation animals: 315 F2 individuals and 186 reciprocal backcross individuals (88 Charolais backcrosses, CB1, and 98 Holstein backcrosses, HB1). Phenotypic scoring for coat colour was performed on the second-generation animals of this population. Seven different subcategories for coat colour were initially defined (Figure 1): White, Off-White, Light-Grey, Grey, Light-Red, Red and Black. This choice of colours was to overcome, as far as possible, misclassification arising from subtle differences in colour and variation resulting from differences in age at scoring. Animals were scored by visual comparison to a colour chart and were photographed. Visual scores were later confirmed using the photographs. Pedigrees were verified using the genotype data. Because it was sometimes difficult to unambiguously assign an animal to one of the colour categories, the following five categories were later defined: White (1; including both White and Off-White animals), Grey (2; Light-Grey and Grey), Light-Red (3), Dark-Red (4) and Black (5). It was on these colour categories that the primary analyses were performed.

Following the approach adopted by Hirooka et al. [32], data from the five-category colour scoring were converted to binary traits, coding as 1 the expression and as 0 the non-expression of each category. Hence, a light-red animal was coded as 0 0 1 0 0 for the five colour categories (White, Grey, Light-Red, Dark-Red and Black). For analysis, we assumed that the Charolais dilution (*Dc*) locus was segregating in this population with the previously-documented mode of inheritance (i.e. with heterozygous animals showing an intermediate level of dilution [17]). Thus the colour data were also condensed into two traits called "Quantitative-Black" and "Quantitative-Red" with the aim of quantifying the Charolais dilution effect on the two types of melanin pigments, eumelanin and phaeumelanin. "Quantitative-Black" included White (1), Grey (2) and Black (3), but excluded the Light-Red and Dark-Red individuals. Quantitative-Red included White (1), Light-Red (2) and Dark-Red (3) individuals. The combined trait, "Quantitative-Dilution," included both black and red pigments: completely diluted animals (White, coded as 1), partially diluted animals (Grey and Light-Red animals, coded as 2) and the absence of dilution effect (Dark-Red and Black, coded as 3).

Following the observation of a wide range of intensities in the diluted categories (Grey and Light-Red), we further defined a binary trait called "Grey-Intensity", which only included the two grey subcategories (Light-Grey, 1, and Dark-Grey, 2), which had been pooled together as "Grey" for the primary analyses.

### Genotyping and Sequencing analysis

The complete population (founders, F1 and second generation cross bred animals) was genotyped for microsatellite markers distributed throughout the whole the bovine genome. DNA was extracted from blood samples [33]. Information from 168 markers was used to build linkage maps for the 29 bovine autosomes using the CRIMAP 2.4 software [34] and the information content (IC) extracted from the linkage maps was obtained according Knott et al. [35]. Genetic maps and their average IC are given [see Additional file 1]. Marker order was in agreement with the latest published version of the bovine linkage map [36].

Genotyping of the whole herd for the E^*D *^and *e *alleles of the *MC1R *gene was performed by KBiosciences (Herts, UK) using a competitive-allele-specific PCR system (KASPar technology). The absence of any of the two tested alleles was considered indicative of the presence of any of the Agouti-responsive alleles found in wild-type colour cattle (*E*^+^).

Genotyping of the c.64A>G variant of the *SILV *gene was obtained for most of the second-generation individuals (F2, CB1 and HB1) with available colour scores. The primers used for amplification of exon 1 were 5' ACTGTCAATGAGTAGCAGGATGTC 3', and 5' TGCACCCAAATCTTCATGTG 3' (434 pb fragment size). Restriction digestion with the Enzyme *ScfI *(New England Biolabs) was used to distinguish the allele containing the A nucleotide at the c.64A>G position (where the product was not cleaved by the restriction enzyme, thus yielding a single band) and the G-containing allele (where the restriction site is present and two bands of 244 and 189 bp are generated). The linkage map of chromosome 5 was rebuilt to include the dinucleotide marker *SILV *c.64A>G, and subsequently, the *Dc *locus presumed genotypes based on phenotypic colour scores (Black and Dark-Red: *dc*^+^*dc*^+^; Grey and Light-Red: *Dcdc*^+^; White: *DcDc*) [17]. The genotypes of the founders for both loci were inferred based on the assumption of a fixed difference between the breeds (i.e. Charolais fixed for "A" and *Dc*, Holstein fixed for "G" and *dc*^+^). A CHROMPIC analysis [34] was performed to identify unlikely double recombination events that might indicate errors in genotyping or phenotype classification.

The coding region of the bovine *SILV *gene was sequenced in 16 second-generation individuals with different coat colours. Primers to amplify the eleven exons of the bovine *SILV *gene were designed on the basis of the gene mRNA sequence, [GenBank: EF065525], and the complete DNA sequence of the gene, [GenBank: NC_007303], based on the bovine genome sequence assembly (Build 3.1) [21]. A pair of sequencing primers was used for each exon, with the exception of exon 6, for which three primers pairs were designed [see Additional file 2]. The PCR products were sequenced using the ABI PRISM Dye Terminator Cycle Sequencing Kit and loaded onto an ABI PRISM 3730 DNA Sequencer. The sequences were checked, aligned and compared using the BioEdit program [37].

### Statistical Analysis

Using the five-category dataset (White, Grey, Light-Red, Dark-Red and Black), the observed proportions of individuals included in each of the classes were compared with those calculated under the hypothesis of fixation of alternative alleles in the founder lines (Charolais genotype: *Dc*/*Dc*; Holstein genotype: *dc*^+^/*dc*^+^) using χ^2^-tests assuming that heterozygotes at the Charolais dilution locus showed intermediate coat colour [17]. The distributions of frequencies in the three classes of genetic backgrounds (CB1, HB1 and F2) were tested separately. The CB1 and HB1 groups were tested against the predicted distributions of 1:1 (white:pale) and 1:1 (dark:pale) ratios, respectively (1 d.f.). The distribution of the F2 individuals was tested across all three dilution phenotype categories (1:2:1, dark:pale:white; 2 d.f.). The same method was used to test the proportions of genotypes obtained for the *SILV *c.64A>G mutation in the three genetic groups, against the hypothesis of fixation of alternative alleles in the founder lines. The effects of experimental variables on coat colour was also investigated using residual maximum-likelihood analysis (REML [38]) implemented within GenStat [39]. This method was also used to study the association of the *SILV *c.64A>G allelic variant with the coat colour dilution variation observed in the resource population.

The primary regression analysis used the linkage map obtained from microsatellite data and assuming the founder lines to be fixed for alternative alleles at the *Dc *locus [40]. It was performed using QTL Express [41] for the combined traits: Quantitative-Dilution, Quantitative-Black and Quantitative-Red. For all traits, a single QTL model with additive and dominance effects was fitted to the data at every centi-Morgan along the chromosome, considering the genetic background (F2, CB1 and HB1) as a fixed effect. For each chromosome, the F-ratio and QTL effect were calculated at the position where the statistic profile reached its maximum. The additive component of the QTL effect was estimated as half of the phenotypic difference between the homozygotes for the Holstein and Charolais alleles. A positive value of the additive effect denotes an increased expression of the coat colour phenotype as a result of the Holstein allele. The dominance effect was calculated as the deviation of the heterozygote from the mean of the two types of homozygous animals. Where the sign of the dominance effect is the same as the additive effects, the Holstein allele was dominant over the Charolais allele, whereas if the signs are opposite, the Charolais allele was the dominant allele. The analysis of Quantitative-Dilution was performed including the *MC1R *genotype (*E*^*D*^-, *ee*, *eE*^+^) as a fixed effect. To avoid the mix of colour backgrounds in the analysis of the two pigment-specific traits, Quantitative-Black and Quantitative-Red, the White animals included in the analysis were selected according their *MC1R *genotype (i.e. animals with *ee *genotype were excluded from Quantitative-Black, and animals with *E*^*D*^- were excluded from Quantitative-Red). To test for a direct relationship of the *SILV *c.64A>G mutation [19] on the linkage associations identified on chromosome 5 for all the dilution-related traits, the genome scan analysis was repeated including the *SILV *c.64A>G genotype as a fixed effect in the regression model. The trait Grey-Intensity was later analyzed, using the *SILV *c.64A>G genotype as fixed effect (because the dataset included both AG and AA individuals).

Permutation testing (10,000 shuffles) was used to obtain the 5% and 1% chromosome-wide and genome-wide thresholds [42]. The 95% confidence intervals (CI) of the significant associations were estimated by bootstrapping [43].

## Authors' contributions

BG-G carried out part of the genotyping experiments, performed the statistical analyses, performed error-checking on phenotype and genotype data, and drafted the manuscript. PW participated in the design and coordination of the study, compiled the phenotype data and helped to draft the manuscript. JLW conceived of the study, participated in its design and coordination, selected the initial marker panel and helped to draft the manuscript. All authors read and approved the final manuscript.

## Supplementary Material

Additional file 1 Linkage map details.Marker positions (cM Kosambi) are shown for the sex-average maps built for the Charolais × Holstein population considered in this study. The average information content (IC) for each linkage group is also indicated. For chromosome 5, the map including the *SILV *c.64A>G mutation is also presented (*). Click here for file

Additional file 2 Primers used for sequencing analysis. Click here for file
