# Supplementary material for: Genetic effects on coat colour in cattle: dilution of eumelanin and phaeomelanin pigments in an F2-Backcross Charolais × Holstein population
Source: BMC Genet. 2007 Aug 16;8:56. doi: 10.1186/1471-2156-8-56 (PMC1994163; doi:10.1186/1471-2156-8-56)
Supplement: Additional file 1 — Linkage map details.Marker positions (cM Kosambi) are shown for the sex-average maps built for the Charolais × Holstein population considered in this study. The average information content (IC) for each linkage group is also indicated. For chromosome 5, the map including the SILV c.64A>G mutation is also presented (*). [file 1471-2156-8-56-S1.doc]

| Chr.  (IC) | Markers and positions (cM Kosambi) | Chr.  (IC) | Markers and positions (cM Kosambi) |
| --- | --- | --- | --- |
| Chr.1  (0.57) | BM6438 0.00 TGLA49 3.3, BMS4017 37.0, TGLA57 50.9, INRA128 70.5, BM864 89.8, CSSM19 119.0, BMS4044 142.9 | Chr.15 (0.59) | BR3510 0.0, JAB1 20.6, BSM2684 32.4, IDVGA10 38.1, DIK2634 60.0 BMS429 80.7 |
| Chr.2  (0.57) | TGLA431 0.0, CSSM42 32.6, BM4440 54.9, TGLA226 81.2, BM2113 114.5, IDVGA2 124.3 | Chr.16  (0.61) | BM121 0.0, TGLA53 12.0, ETH11 27.8, BM719 55.8, BM1706 63.4, HUJ625 75.4, DIK4011 87.5 |
| Chr. 3  (0.65) | ILSTS96 0.0, TGLA263 24.1, INRA123 37.3, INRA130 37.5, IOBT250 52.5, HUJ1177 63.6, IDVGA35 76.1, IDVGA27 98.7 | Chr.17  (0.59) | URB48 0.0, BMS1373 28.6, TGLA231 41.8, IDVGA40 50.7, INRA25 76.5, BM1233 89.1 |
| Chr.4  (0.53) | BMS1788 0.0, MAF50 62.3, DIK26 92.3, IDVGA51 96.1, RM88 112.5, MGTG4B 128.3 | Chr.18  (0.59) | IDVGA31 0.0, ABS013 17.7, INRA121 30.9, HAUT14 43.2, DIK67 69.0 |
| Chr.5  (0.66) | BM6026 0.0, RM103 17.8, DIK4136 28.6, BM321 33.7, DIK4782 41.3, BMS1617 53.8, BR2936 62.4, ETH10 66.3, DIK5248 73.2, IGF1 81.1, DIK5104 91.4, ILSTS034 110.5, ETH152 129.6 | Chr.19  (0.58) | HEL10 0.0, BMS2142 27.6, CSSM65 53.5, ETH3 72.3 |
| Chr.5*  (0.68) | BM6026 0.0, RM103 18.2, DIK4136 28.4, BM321 33.3, DIK4782 40.6, BMS1617 53.0, BR2936 61.5, ETH10 65.3, *SILV*c.64A>G 67.3, DIK5248 72.3, IGF1 80.3, DIK5104 90.9, ILSTS034 109.5, ETH152 128.5 | Chr.20  (0.59) | BM3517 0.0, TGLA126 31.4, DIK15 46.1, BM5004 66.7 |
| Chr.6  (0.65) | DIK5076 0.0, BM1329 32.6, DIK1054 45.9 DIK82 56.3, DIK2320 65.8, CSN3 81.1, BP7 90.5, DIK1180 106.6, BMS739 109.5, BM2320 133.3 | Chr.21  (0.36) | HEL5 0.0, TGLA337 69.4, IDVGA69 84.8 |
| Chr. 7  (0.49) | BP41 0.0, RM6 9.5, BM1853 53.9, ILSTS6 84.0, INRA53 93.5 | Chr.22  (0.59) | DIK1161 0.0, BMS742 19.5, BM3406 28.5, BM3628 41.8, DIK2443 48.8, HAUT24 59.9, UWCA49 70.4, DIK5307 83.5 |
| Chr.8  (0.52) | IDVGA11 0.0, DIK106 19.7, HUJ174 57.0 HEL9 80.1, DIK74 97.4, CSSM47 119.0 | Chr.23  (0.41) | IOBT528 0.0, BMS2268 80.3, BM1905 87.5 |
| Chr.9  (0.58) | ETH225 0.0, BM2504 16.6, UWCA9 40.5, MM12E6 68.8, INRA84 77.9 | Chr.24  (0.57) | TGLA351 0.0, CSSM23 11.1, ILSTS101 28.2, INRA90 49.7 |
| Chr.10  (0.63) | DIK5169 0.0, BMS528 26.9, TGLA378 53.7, BM888 66.4, CSRM60 87.8, MNB78 96.1, TGLA272 113.9, CSSM46 122.3, BL1134 133.2 | Chr.25  (0.65) | BM4005 0.0, BM737 17.5, INRA222 34.6 |
| Chr.11  (0.55) | BM716 0.0, INRA177 17.0, ILSTS100 35.1, IDVGA3 63.6, HUV174 74.6, BMS607 89.6 | Chr.26  (0.62) | ABS12 0.0, HEL11 13.5, RM26 28.6, IOBT730 38.9 |
| Chr.12  (0.64) | BMS410 0.0, BMS2057 26.4, RM162 43.8, BM6404 57.2, DIK4028 68.7, INRA5 82.2, URB054 94.6, INRA209 109.9 | Chr.27  (0.48) | BM3507 0.0, RM209 15.8, BM203 64.6 |
| Chr.13  (0.60) | HUJ616 0.0, DIK54 10.9, ABS10 33.2, DIK93 51.2 | Chr.28  (0.69) | BP23 0.0, IDVGA43 19.3, BMS2658 26.9, IDVGA8 34.5 |
| Chr.14  (0.62) | CSSM66 0.0, RM11 34.2, PZ271 58.0, BM4513 68.3, BM2934 73.7 | Chr.29  (0.67) | TGLA26 0.0, RM44 21.0, MNB166 31.4, DIK94 39.7, MNB101 67.6 |
