# Supplementary material for: Genetic effects on coat colour in cattle: dilution of eumelanin and phaeomelanin pigments in an F2-Backcross Charolais × Holstein population
Source: BMC Genet. 2007 Aug 16;8:56. doi: 10.1186/1471-2156-8-56 (PMC1994163; doi:10.1186/1471-2156-8-56)
Supplement: Additional file 2 — Primers used for sequencing analysis. [file 1471-2156-8-56-S2.doc]

SILV_ex1seq_fwd: GTTCTTTCTCAGGTCCTTCTGC

SILV_ex1seq_rev: TGCCCATGTCCTCACTAAATC

SILV_ex2seq_fwd: CTGACCTCACATGCCACAAC

SILV_ex2seq_rev: TATGTGGCCACCTGAAAAGG

SILV-ex3seq_fwd: TTTCCAGGGAGGATATGGTG

SILV-ex3seq_rev: TGTCCACTGGGTCTTCTGTG

SILV-ex4seq_fwd: CCCTTCTACCACTCCAAATAGG

SILV-ex4seq_rev: AGGTGCTGTGGAAGAGGAAG

SILV_ex5seq_fwd: CATCCTTGATACATCTCCTGACC

SILV_ex5seq_rev: CCCACATCTTCCTGGTTCTG

SILV_ex6Aseq_fwd:CAGAACCAGGAAGATGTGG

SILV_ex6Aseq_rev:GCAGTTGTCACATGCCTATC

SILV_ex6Bseq_fwd: ACCTGGGACTTTGGTGACAG

SILV_ex6Bseq_rev: ACCAGCTCTGGAGTTGTTCC

SILV_ex6Cseq_fwd: GCCAACCACAGAGGATGTAG

SILV_ex6Cseq_rev: AGTGAAGATTCTGAGGGGACTC

SILV_ex7seq_fwd: GGGGCAGTCATTTCTCTTG

SILV_ex7seq_rev: TATGAAACCCCTGGGATCTG

SILV_ex8seq_fwd: CCTGGACAAGGAGAATACCC

SILV_ex8seq_rev: CGATGAGATGTCCATGCAG

SILV-ex9seq_fwd: TGCATTTGAGCTGACTGTGTC

SILV-ex9seq_rev: TTGCCCTATGGGTGGAATC

SILV_ex10seq_fwd: GCCTGGTAGGTAGTTGGACAAG

SILV_ex10seq_rev: CTGGGCTCTTTCTTGGACTC

SILV_ex11seq_fwd: GACCAAGTCAACCTGGGTTATG

SILV_ex11seq_rev: TTTTCCCCACTGGGATAGC
